# Developing an Integrated Medical-Veterinary Data Framework for Investigating Human Toxoplasmosis: A One Health Perspective

**DOI:** 10.3390/pathogens15020210

**Published:** 2026-02-13

**Authors:** Anna Maria Fausta Marino, Renato Paolo Giunta, Antonio Salvaggio, Vincenzo Agozzino, Alessandra Aparo, Maurizio Percipalle

**Affiliations:** Italian National Reference Center for Toxoplasmosis, Istituto Zooprofilattico Sperimentale della Sicilia, 95125 Catania, Italy; renato.giunta@izssicilia.it (R.P.G.); antonio.salvaggio@izssicilia.it (A.S.); vincenzo.agozzino@izssicilia.it (V.A.); alessandra.aparo@izssicilia.it (A.A.); mpercipalle@gmail.com (M.P.)

**Keywords:** toxoplasmosis, epidemiological investigation, One Health

## Abstract

Toxoplasmosis is an insidious globally distributed zoonosis accounting for approximately one-fifth of all foodborne illnesses in humans in Europe. It stands as a classic example of a disease transmitted through the close interconnection between humans, animals, and the environment. Accordingly, mitigation strategies and health management protocols demand cross-sectoral involvement from medical, veterinary, environmental, and political actors, rendering the adoption of a ‘One Health’ perspective essential. Despite longstanding advocacy for One Health by the WHO, WOAH, and FAO, national health authorities have yet to establish the necessary operational infrastructure. Specifically, there is a lack of tools to enable information sharing among professionals, which is essential for the synergistic management of major health issues. A four-part epidemiological data collection tool specifically developed for human toxoplasmosis is proposed here to aid in the identification of risk factors and potential sources of infection. The proposed framework comprises four sequential sections intended to be completed through contribution from the patient, the attending physician, the involved veterinarian, and the national reference laboratory for toxoplasmosis. This questionnaire serves as a conceptual, non-validated tool designed to support a One Health–oriented epidemiological investigation process. Its practical performance, feasibility, and potential usefulness in routine surveillance or prevention strategies have not yet been assessed and will require validation in future studies. Nonetheless, the framework may serve as a model for the development of similar integrative tools applicable to other zoonotic diseases.

## 1. Introduction

In our global era, the COVID-19 pandemic is a dramatic reminder of how human, animal and environmental health are deeply intertwined. Treating humans, animals, and the environment as separate compartments undermines effective responses to emerging zoonotic threats, as the understanding and control of dangerous zoonoses are inherently shaped by their interactions. Disease emergence is influenced by a variety of interconnected factors, including increased human activity in biodiversity-rich areas, increased contact between humans, livestock, and wildlife due to habitat loss, climate change altering transmission dynamics, and the continued use and trade of unconventional wildlife species in close proximity to domestic animals.

In the current context, preventing and controlling zoonoses necessitates the “One Health” framework, as defined over the years by the World Health Organization as “an approach to designing and implementing programs, policies, legislation and research in which multiple sectors communicate and work together to achieve better public health outcomes.” One Health has also been defined by the One Health Commission as “a collaborative, multisectoral, and trans-disciplinary approach—working at local, regional, national, and global levels—to achieve optimal health and well-being outcomes recognizing the interconnections between people, animals, plants and their shared environment” [1].

Although rarely making headlines, toxoplasmosis is a quintessential One Health challenge. Unlike many other pathogens shared between humans and animals, *Toxoplasma gondii* is ubiquitous, found all over the world, and can infect all warm-blooded animals. Transmission can occur through food of both animal and vegetable origin, or via environmental exposure to oocysts shed in cat feces, making source attribution essential for prevention.

Globally, toxoplasmosis is the most prevalent foodborne parasitic zoonosis. It ranks third among all foodborne pathogens in the United States [2] and second in Europe [3]. According to the World Health Organization, *T. gondii* accounts for roughly one-fifth of all human foodborne diseases in Europe, affecting more than one million people annually. In the Netherlands alone, the societal burden is estimated at €45 million per year in disability-adjusted life years (DALYs) and direct costs [4].

While most infections are asymptomatic or self-limiting, clinical toxoplasmosis can cause severe outcomes ranging from miscarriage in pregnant women, birth defects in newborns and ocular and neurological disease, especially in immunosuppressed individuals. In livestock, the parasite causes reproductive losses that directly affect farm profitability and indirectly impact the food industry. Without biosecurity measures at the primary production level—often costly and difficult to sustain—food producers cannot ensure safe products for vulnerable consumers. Despite these risks, routine testing for *T. gondii* in meat is not required under most national or international food regulations, including in the European Union.

Since its discovery in the beginning of the last century (1908) and the first case attributed to *T. gondii* identified more than 80 years ago (1939), substantial advances have been made in both veterinary and medical research. However, cross-sector collaboration has lagged, limiting the integration of knowledge about the parasite’s biology, ecology, and host–environment interactions. Current control measures focus mainly on mitigating consequences for infected hosts rather than addressing the complex interaction of the parasite with environmental and hosts biological factors. A broader, integrated approach involving veterinarians, physicians, biologists, epidemiologists, and environmental scientists is needed to develop cost-effective and efficient prevention and tracing tools.

While private and public animal health professionals have fully embraced the One Health concept, prioritizing consumer health above animal health and welfare, which are the cornerstones of veterinarians’ commitment, a significant challenge remains. There is still mistrust and skepticism among medical doctors regarding a shared approach to preventing and managing health emergencies. This would prove especially useful for diseases like toxoplasmosis, as treatment is not always effective particularly if it is not started promptly. Consequently, control strategies for toxoplasmosis depend entirely on prevention at every stage of transmission, from the environment to the human host.

Accurate source attribution is central to such an approach. If infection originates from undercooked meat, interventions may include strengthening food safety regulations, improving hygiene condition at the primary production level, enhancing meat inspection, and promoting safe cooking practices. If oocyst contamination is the primary source, preventive measures should focus on water protection, soil management, and safe handling of cat feces.

While prenatal screening is crucial for preventing congenital toxoplasmosis, we must broaden our approach to understanding how toxoplasmosis spreads. Owing to the marked variability in sources of infection, we should also extend our investigation of the disease’s spread and its risk factors to include other professional categories who might encounter the parasite as an occupational hazard. Notably in food, *Toxoplasma gondii* has been detected not only in uncooked meat, particularly pork and lamb, but also in raw produce and, less frequently, in fresh fish and seafood [5].

In Europe, the surveillance of *T. gondii* is inconsistent. Monitoring is only mandated under Directive 2003/99/EEC [6] when epidemiologically justified, resulting in fragmented data. EFSA and ECDC agree that human toxoplasmosis is both underdiagnosed and underestimated, and that outbreaks—often linked to contaminated water—are rarely detected due to the parasite’s long incubation periods, frequent subclinical infections, and delayed symptom onset. A Europe-focused ranking exercise, using multicriteria decision analysis, lists *T. gondii* among potentially food-borne parasites of importance, and that are currently not routinely controlled in food [7].

Effective prevention demands moving from a reactive to a proactive approach. Epidemiological tools can function as early warning systems, particularly when environmental conditions—such as heavy rainfall facilitating oocyst runoff—favor parasite spread. This enables timely public health responses, including targeted advisories, strengthened food safety protocols, and control measures before outbreaks escalate.

The severity of disease is another issue to take into account, as it depends on multiple factors, including parasite strain, infectious dose, host immune status, and, in congenital cases, timing during pregnancy. There is also experimental evidence that infections from oocysts may cause more severe disease than those from tissue cysts [8]. The main challenge in tracing the source of infection is that, soon after ingestion, the parasite rapidly transforms into a fast-multiplying tachyzoites. Once inside the host, the parasite’s appearance and behavior are largely the same, regardless of whether it initially came from oocysts (sporozoites) or cysts (bradyzoites). Therefore, determining the likely source of infection in an individual case merely relies on epidemiological investigation (e.g., patient’s dietary habits, exposure to cat feces, travel history, outdoor activities) rather than specific laboratory markers that distinguish between cyst and oocyst origins. These uncertainties highlight the need for a comprehensive, One Health-driven research and prevention strategy. By integrating epidemiological insights with veterinary, environmental, and medical expertise, we can better understand *T. gondii* transmission dynamics and design interventions that address all stages of the human–animal–environment interface.

Medical professionals acknowledge the significance of a One Health approach in tackling zoonoses but, they struggle with lack of dependable tools for seeking effective preventive strategies. While these tools should be uncomplicated and straightforward, they must be designed, disseminated, and field-tested to be evaluated. It is the responsibility of scientific committees of global and national health agencies, including the World Health Organization (WHO), the World Organization for Animal Health (WOAH) and national reference laboratories for foodborne diseases, to develop these solutions, which must then be implemented by the appropriate health authorities, with input of frontline health practitioners.

Ultimately, leveraging epidemiology helps us shift from merely reacting to outbreaks to adopting a more strategic, preventive approach to managing toxoplasmosis.

This study aims to offer a structured, epidemiological questionnaire designed to support source attribution of human toxoplasmosis within a One Health framework, and to discuss its possible implementation and applicability to other zoonoses characterized by human–animal–environment interactions.

## 2. Epidemiological Survey Design and Methods

Since the activities and strategies to foster a One Health approach to public health and food safety are the responsibility of the reference centers for foodborne diseases established by European and national public health authorities, we developed a four-part multi-source epidemiological questionnaire in the form of a joint case record that medical professionals, veterinarians, and laboratory scientists can collaboratively complete, deploying their respective expertise (Appendix A). The primary goal of this questionnaire is to conduct a source investigation for a laboratory-confirmed case of toxoplasmosis. It aims to trace back the likely source of infection (foodborne or environmental) by gathering extensive data on the patient’s habits, environment and exposure likely leading to infection.

The questionnaire contains close-ended questions with limited answer choices, either yes/no or multiple-option selections. Open-ended fields are also available for short descriptions filled in by official veterinarians about the hygienic conditions of slaughterhouses and meat production plants as well as food business operators’ compliance with European food legislation. In addition, the questionnaire also collects laboratory data relating to the identification and genotyping of *T. gondii* isolates.

Since the questionnaire is intended to be administered only to sick or infected subjects, the major goal of the epidemiological investigation is to pinpoint and estimate the main risk factors for infection.

All data are collected anonymously and stored according to the current EU General Data Protection Regulation (EU-GDPR) 2016/679. Data governance arrangements—including hosting solutions, data access policies, and the practical application of GDPR requirements—will necessarily depend on the legal, organizational, and technical frameworks of the competent national or EU authorities adopting the questionnaire, leaving operational, legal, and infrastructural implementation to the responsible authorities.

The questionnaire’s opening section (part I) is dedicated to the patient’s personal and exposure history. This section can be self-administered by the patient or completed collaboratively with the attending physician who is also required to sign this document to validate its contents. Data collected includes dietary habits (e.g., consumption of undercooked meat, unwashed fruits and vegetables), occupational risks (such as farming, veterinary work, or laboratory exposure), and travel or lifestyle behaviors (such as outdoor activities or contact with cats) that may increase the likelihood of contact with *T. gondii*. This section establishes the baseline for identifying potential routes of infection and for linking clinical findings and laboratory data with potential environmental or animal sources.

The effectiveness of this section of the questionnaire is dependent on the motivation of the patient to provide accurate and detailed retrospective information. Recalling specific dietary habits, pet husbandry practices, and environmental exposures over a relevant period requires significant patient engagement. Thus, securing informed consent and clearly communicating the direct public health benefit of their contribution—namely, helping in source identification to prevent further infections—are essential steps to foster cooperation and ensure the reliability of the data for a thorough exposure assessment.

The second section of the questionnaire (part II) must be filled out by the reporting physician and collects clinical, diagnostic, and epidemiological data. Information recorded includes physician identification, patient demographics (age, sex, residency, pregnancy status) laboratory confirmation of toxoplasmosis (serology, PCR, histology, imaging), medical history (co-morbidities, transfusion, or transplant history), and treatments administered. Physicians are also asked to indicate the suspected source, presumed period and place of infection.

The third section of the questionnaire (part III) must be completed by an official veterinarian. This part is designed to investigate potential sources of *Toxoplasma gondii* infection, specifically those related to food or contact with cats. Information gathered included presumed foodborne exposure (suspected contaminated food matrices, place of purchase or consumption, traceability of products, and evaluation of food safety practices along the supply chain), waterborne exposure (source of water, certification of potability, use for consumption or irrigation, and control measures against contamination), and environmental exposure (diagnostic testing of animals owned by the patient, animal feed, and environmental samples). Where relevant, sampling reports and laboratory results are recorded to support source attribution. The purpose of this section is to identify upstream risk factors at the human–animal–environment interface.

The fourth section of the questionnaire (part IV) falls under the responsibility of the Italian national reference center for toxoplasmosis (Ce.Tox) and addresses analytical confirmation and genotyping of *Toxoplasma gondii* strains. This involves analyzing the parasite’s DNA from human, animal, and food samples submitted with corresponding sampling reports. Test results provide confirmation of infection and, when possible, molecular characterization of the *T. gondii* strain involved. A summary of laboratory findings is prepared and attached to the case record. This summary is then integrated into the epidemiological survey, which strengthens the investigation by linking clinical, veterinary, and environmental data to aid in source attribution and strengthen the overall investigation.

Given the preliminary nature of this study, it should be noted that the questionnaire presented here is an unvalidated prototype. Standard validation criteria were not applied, as the tool is currently conceptual and intended to suggest a structured framework for data collection and source attribution in toxoplasmosis. Future studies are needed to evaluate its practical performance, refine its content, and validate its use across different epidemiological settings.

## 3. Discussion

Epidemiological questionnaires are crucial for tracking the spread of infectious diseases, especially those with high transmissibility and a high R0 coefficient, like COVID-19 or influenza. In such cases, contact tracing and isolating infected individuals are essential measures to contain outbreaks and limit further transmission [9].

However, the utility of these questionnaires extends to other diseases such as toxoplasmosis, presenting unique challenges for epidemiological investigation. Unlike highly transmissible respiratory pathogens, *Toxoplasma gondii* infections are often asymptomatic or result in mild, nonspecific symptoms, while acute clinical presentations are relatively uncommon. As a result, a substantial proportion of infections remain undiagnosed and clusters of cases—particularly acute syndromes linked to a common source—have only rarely been documented [10]. This consistent underdiagnosis has historically hampered efforts to establish accurate epidemiological patterns and has contributed to underestimation of the true disease burden.

On the other hand, unlike emerging viral zoonoses, which rely on specific conditions to cross and adapt between diverse host species, the transmission dynamics of *T. gondii* among animals, humans, food, and the environment are well understood, allowing interventions to target these points to disrupt the cycle. Achieving this goal could benefit from the adoption of new investigative approaches. Specifically, a standardized questionnaire could aid attribution of infection sources, support the development of local epidemiological datasets that capture prevalence and transmission dynamics, and provide evidence to drive interventions targeting specific transmission routes at all levels (food businesses, farms, households). Standardized epidemiological questionnaires have long been validated and successfully used in toxoplasmosis research, particularly in case–control and multicenter studies, to identify key risk factors such as consumption of undercooked meat, soil contact, and environmental exposure. European multicenter studies on acute toxoplasmosis in pregnancy [11] and national investigations, including those conducted in the Netherlands [12], have demonstrated that well-designed questionnaires can provide robust and actionable insights into likely transmission pathways, even in the absence of molecular source markers. However, most existing tools remain limited to single-sector data collection and are rarely applicable to sporadic toxoplasmosis cases because molecular attribution is difficult and exposure histories are fragmented.

Our investigation, the first of its kind in Italy, is novel in its structured integration of medical records, veterinary findings and laboratory results, embodying a One Health approach to source attribution and prevention. A coordinated One Health strategy integrating human, animal, and environmental health sectors, is vital for effective toxoplasmosis control [13]. By linking clinical manifestations with potential zoonotic and environmental exposures, the epidemiological investigation enhances the likelihood of correctly attributing infection sources, an area that has traditionally been a major gap in toxoplasmosis epidemiology either inside or outside the EU. Rather than identifying new risk factors, its novelty lies in its cross-sectoral integration and standardized structure, facilitating comparability across cases and providing a common starting point for future validation studies.

A scientific opinion published in 2018 by the European Food Safety Authority (EFSA) highlights that *Toxoplasma gondii*, together with *Echinococchus* spp. and *Cryptosporidium* spp., is not routinely monitored as part of official controls in food production, and that under-reporting of cases persists despite evidence indicating a considerable human health burden. The opinion further notes that a significant proportion of infections are attributable to foodborne transmission, particularly through the consumption of undercooked meat and contaminated fresh produce, although the relative importance of different transmission routes remains uncertain [7]. Nonetheless, *T. gondii* is included among the zoonotic agents to be reported in the EU One Health Zoonoses Reports which are published annually by the EFSA and the European Centre for Disease Prevention and Control (ECDC) in accordance with the EU directive 2003/99/EC on the monitoring of zoonoses and zoonotic agents [14]. Under these regulations, *T. gondii* is classified in List B, encompassing agents whose reporting is conditional on the epidemiological situation; thus, mandatory reporting occurs only when public health circumstances warrant it. This conditional and often incomplete reporting, particularly the underreporting of non-congenital toxoplasmosis, hampers a comprehensive understanding of the true prevalence of the disease across Europe. As a result, knowledge of toxoplasmosis remains fragmented, and accurately assessing the risk associated with foodborne transmission is challenging. In contrast, congenital toxoplasmosis benefits from more consistent documentation through national monitoring programs.

This fragmented knowledge likely stems from the disease’s inherent complexities, including its often-sporadic nature and the substantial challenges in conducting trace-back investigations. Crucially, the success of any surveillance or risk-management system relies on high-quality patient-derived data, which are difficult to collect without robust protocols and, most importantly, sustained patient compliance in providing detailed retrospective exposure history. The lack of documented efforts in the scientific community thus reflects not a lack of concern, but the difficulties in implementing effective surveillance and source attribution for this specific zoonosis.

A standardized questionnaire for the epidemiological investigation of toxoplasmosis is therefore a useful tool in addressing the substantial knowledge gaps that currently hinder effective surveillance of the zoonosis in Europe. By allowing the systematic collection of comprehensive exposure histories, including dietary habits, food handling practices, interaction with domestic and wild animals, environmental exposures, and travel history, a harmonized, patient-focused questionnaire helps in overcoming these restrictions [15]. This information is critical in determining the relative contribution of different routes of infection, such as ingestion of tissue cysts in undercooked meat, oocyst contamination of fresh produce and water, or direct contact with contaminated soil. For example, recent reviews show changing patterns in outbreak sources over time: earlier decades (1960s–1990s) often implicate undercooked meat or meat derivatives; later decades show increasing importance of oocyst transmission via water, soil, and fresh produce [16].

Similar approaches have proven highly effective in the surveillance of other foodborne zoonoses. Standardized outbreak investigation questionnaires, for example, have been crucial in identifying the origins of *Salmonella* [17] and *Listeria monocytogenes* [18] infections throughout the European Union providing the evidence base for targeted risk management interventions such as control programs in poultry and the implementation of stricter food hygiene measures. Likewise, case–control studies of campylobacteriosis, which are frequently supported by structured questionnaires, have shed light on the roles of poultry consumption and cross-contamination in the domestic kitchen leading to risk-based control strategies [19]. These examples show how methodical patient-derived data collection can enhance risk assessment and public health policy while providing useful insights into transmission pathways.

Using a similar approach for toxoplasmosis would enhance source attribution models, increase cross-country comparability, ultimately allowing for a more accurate estimation of its prevalence thereby implementing targeted prevention effectively. This would therefore make it easier to plan more effective risk-reduction strategies, such as consumer education on safe meat preparation, improved monitoring of fresh produce, or making targeted changes to livestock production systems. Ultimately, the implementation of a standardized epidemiological questionnaire for toxoplasmosis cases could transform fragmented surveillance efforts into a coherent framework, thereby enhancing Europe’s capacity to monitor, prevent, and control this important zoonosis [20].

While standardized questionnaires and case–control studies have proven highly effective for zoonotic enteric pathogens, their application to toxoplasmosis faces notable challenges. The sporadic occurrence of cases, long incubation period, and unreliable recall of exposures reduce their value for source attribution of *Toxoplasma gondii*. Moreover, their success depends on case detection, which is hindered by the predominantly silent or subclinical course of infection. Reliance on patient recall for dietary or environmental exposures introduces the risk of recall bias while the feasibility of veterinary and laboratory follow-up is contingent not only on resources and infrastructure but also on the timely reporting of clinical cases and the accurate recording of patients’ exposure histories. These shortcomings can be mitigated through systematic screening in high-risk groups (e.g., pregnant women, immunocompromised patients), or by incorporation of standardized exposure checklists to reduce recall errors.

In livestock production systems and short food chains, failures to implement correct biosecurity practices—especially those targeting *T. gondii* containment—are typically not isolated incidents. Consequently, these deficiencies can be identified, even retrospectively, to facilitate the timely application of corrective measures. The efficacy of the expected outcome is directly proportional to the promptness of the epidemiological investigation conducted by the involved professionals and stakeholders.

A further critical limitation relates to laboratory confirmation. Although molecular methods and strain genotyping are powerful tools for source attribution, their use is constrained by several factors: the availability and quality of biological samples, the timing of collection in relation to the infection, and the accessibility of equipped laboratory facilities. In many cases, human, food, or animal samples may be insufficient, degraded, or not collected at all, which hampers the likelihood of obtaining a definitive genetic fingerprint of the parasite. Even when molecular testing is available, disparities in laboratory capacity between regions can restrict its routine implementation. These limitations may ultimately hinder the tool’s ability to provide conclusive evidence linking human cases to specific sources of infection. To overcome the limitations associated with molecular confirmation and strain genotyping, several strategies can be implemented. Standardized protocols for the collection, handling, and transport of biological (e.g., blood, amniotic fluid, tissue biopsies), food, and environmental samples, coupled with training on optimal timing of sampling, would improve the availability and quality of material for testing. Establishing biobanks to store human, animal, and food samples would also enable delayed or retrospective molecular analysis when immediate testing is not feasible. Systematically linking molecular results with medical, veterinary, and environmental data gathered through the questionnaire would enhance the value of genotyping for source attribution and also for revealing the emergence and tracing of clonal lineages that are more common in other parts of the world (especially in South America) or that exhibit a higher degree of pathogenicity. This is especially true for atypical strains that are currently believed to be responsible for clinical disease even in immunocompetent subjects. Finally, participation in international *T. gondii* strain databases and collaborative networks would promote comparability of results, facilitate outbreak investigations, and strengthen global surveillance.

Historically, collaboration between the medical and veterinary fields has been fragmented, leading to misattribution of infection sources. A medical-only approach focuses primarily on clinical outcomes and patient history, but it cannot adequately trace environmental or animal sources. Conversely, a veterinary-only perspective may identify infection dynamics in animals but fails to capture the human health impact or patient-level exposures. Ultimately, while laboratory diagnosis provides critical insight into the pathogen’s molecular fingerprint, its full interpretative value is limited unless integrated with high-quality epidemiological and veterinary data. This disconnection has probably contributed to missed opportunities in identifying critical control points for prevention.

Another important outcome of the questionnaire is the potential to provide benefits to public health policies. By systematically collecting data, the tool can generate robust epidemiological evidence that helps clarify the relative importance of the main transmission routes, whether through undercooked meat, contaminated water, soil exposure, or contact with cats. This is particularly valuable given the often-asymptomatic nature of infection and the long latency period, which can otherwise obscure the pathways of transmission.

Beyond generating scientific insight, the tool can assist public health authorities in risk assessment activities since this evidence-informed approach facilitates the targeted allocation of preventive measures. Where collected data indicate that consumption of undercooked meat is the predominant cause of infection in certain areas, authorities could prioritize resource allocation to enhance meat inspection efforts. On the other hand, in settings with a suspected predominance of waterborne transmission, regulatory focus should be placed on surveillance and monitoring of public water supplies. Just the simple act of gathering data on consumption habits, food sourcing, meat preparation, and environmental exposures is instrumental in pinpointing which regulatory measures are more likely to be effective and cost-efficient.

Finally, the consistent use of such tools enables the development of more accurate and customized prevention guidelines for high-risk groups while also revealing knowledge gaps and which prevention messages are poorly known or practiced within a particular sociological or regional context. Advice based on local evidence rather than generalized assumptions might be beneficial especially for vulnerable groups including pregnant women or immunocompromised individuals. Nutrition recommendations, safe food handling, and pet care practices can all be better adjusted when backed by solid region-specific data.

## 4. Conclusions

By merging information across disciplines, the questionnaire for the epidemiological investigation of toxoplasmosis translates One Health principles into practical investigative tools and fosters structured cooperation among professionals from different health domains. When combined with laboratory confirmation and genotyping, this integration may contribute to capturing the complexity of *T. gondii* transmission, while acknowledging that its practical performance has not yet been evaluated.

Within a One Health perspective, the questionnaire represents a conceptual step toward integrated data collection across human, animal, and environmental health sectors. It is designed to be accessible to all healthcare professionals involved in prevention, diagnosis, and treatment of the disease and to facilitate collaborative compilation of information relevant to epidemiological investigation. By linking multi-source data in a single framework, the tool ought to support source attribution, inform public health and food safety policies, although its reliability, feasibility, and added value remain to be assessed through future validation studies. With suitable adaptation and validation, similar frameworks could be considered for other zoonoses.

Even for an underdiagnosed disease with infrequent acute cases, such an integrated approach is essential to reveal hidden transmission routes, quantify risk factors, and reduce the burden of toxoplasmosis.

Without cross-sectoral collaboration, critical pathways remain overlooked, limiting the effectiveness of control measures. A One Health framework therefore provides a relevant conceptual platform for addressing the multifactorial nature of *T. gondii* transmission and to design interventions that integrate human, animal, and environmental health.

In this context, the development of standardized, One Health-oriented epidemiological tools should be viewed as a responsibility of the scientific community. Once rigorously evaluated and formally validated through pilot and validation studies, such tools may help support cross-sectors collaboration, enhance surveillance, and inform preventive measures, potentially reducing the disease impact in both humans and animals. Within this framework, standardized epidemiological questionnaires could ultimately contribute to refined risk assessment and the prioritization of preventive strategies, especially for vulnerable populations.

## Data Availability

The original contributions presented in this study are included in the article. Further inquiries can be directed to the corresponding author.

## Abbreviations

The following abbreviations are used in this manuscript:

WOAH	World Organization for Animal Health
EFSA	European Food Safety Authority
ECDC	European Centre for Disease Prevention and Control
WHO	World Health Organization
FAO	Food and Agriculture Organization of the United Nations

## Appendix A

Epidemiological Investigation Form for Laboratory-Confirmed Clinical and Congenital Toxoplasmosis

PART 1: GENERAL INFORMATION Patient Identification Code: __________________(*to be entered by the Signatory Doctor*) Instructions: To be filled out by the patient or in collaboration with the patient. 1. Social and Demographic Information • City of residence: ___________________ • Marital status: ☐ Married/Cohabitee ☐ Single • Living alone? ☐ Yes ☐ No • Children: ☐ Yes ☐ No ○ Number: _______ ○ Age(s): ___________________ • Pregnant women at home: ☐ Yes ☐ No • Type of housing: ☐ Apartment ☐ Villa with garden ☐ Housing estate ☐ Rural house ☐ Other: ___________ • Education level: ___________________ • Occupation/Job description: ___________________ • Workplace environment: ☐ Office ☐ Countryside ☐ Factory ☐ Ship ☐ Other: ___ • Previous employment history: ______________________________________________ • Home gardening activities: ☐ Yes ☐ No • Home cooking activities: ☐ Yes ☐ No • Smoking habit: ☐ Yes ☐ No • Activities connecting you with nature or animals: ____________________________ • Frequent traveler: ☐ Yes ☐ No ○ Recent locations visited (4–5 places): ___________________________________ • Awareness of Toxoplasmosis: ☐ Yes ☐ No • Awareness of transmission routes: ☐ Yes ☐ No 2. Food Consumption Habits • Water intake: ☐ Tap ☐ Bottled ☐ Urban source • Consumption of raw or undercooked foods: ☐ Yes ☐ No ○ Specify: ________________________________________ • Consumption of ready-to-eat and/or frozen industrial foods: ☐ Yes ☐ No ○ Specify: ________________________________________ • Meat consumption: ☐ Yes ☐ No ○ Raw or undercooked: ☐ Yes ☐ No ○ Kind of meat: ☐ Pork ☐ Lamb/mutton ☐ Beef ☐ Chicken ☐ Rabbit meat ☐ Horse meat ○ Purchase source: ☐ Butcher ☐ Grocery stores • Cured meat products: ☐ Yes ☐ No ○ Specify: ________________________________________ ○ Type: ☐ Fresh ☐ Short-aged ☐ Long-aged • Fruit consumption: ☐ Yes ☐ No ○ Specify: ____________________________ ○ Organic: ☐ Yes ☐ No ○ Home-grown: ☐ Yes ☐ No • Vegetable consumption: ☐ Yes ☐ No ○ Specify: ________________________________________ ○ Organic: ☐ Yes ☐ No ○ Home-grown: ☐ Yes ☐ No ○ Form: ☐ Raw ☐ Fresh ☐ Cooked ☐ Frozen ☐ Packaged • Use of disinfectants for washing produce: ☐ Yes ☐ No ○ Specify products: ________________________________________ • Dairy products: ☐ Yes ☐ No ○ Type: ☐ Fresh ☐ Short-aged ☐ Long-aged • Clams and mussels: ☐ Yes ☐ No ○ Specify: ___________________________ • Raw fish: ☐ Yes ☐ No ○ Specify: ___________________________ • Insect-based foods: ☐ Yes ☐ No ○ Specify: ___________________________ • Occasional consumption of foreign/ethnic cuisine: ☐ Yes ☐ No ○ Specify: ___________________________ • Frequent fast-food consumption: ☐ Yes ☐ No ○ Specify: ___________________________ • Regularly eat at specific restaurants/canteens: ☐ Yes ☐ No ○ Specify: ___________________________ 3. Pets and Animal Contact • Cats at home: ☐ Yes ☐ No ○ Number: _______ ○ Age(s): _______ ○ ID transponder nr.: ___________________ ○ Sterilized: ☐ Yes ☐ No ■ If yes, all cats ☐ Yes ☐ No ○ Indoor only: ☐ Yes ☐ No ○ Diet: ☐ Packaged (dry ☐ wet ☐) ☐ Home-made fresh and raw ☐ Home-made fresh and cooked ☐ Leftovers ○ Litter box use: ☐ Yes ☐ No ☐ Other: __________________ ○ Person in charge of droppings disposal: ____________________ ○ Frequency: ___________________________ • Other animals at home: ☐ Yes ☐ No ○ Species and number: __________________ ○ ID transponder nr.: ___________________________ ○ Diet: ________________________________ • Stray/roaming animals entering premises: ☐ Yes ☐ No ○ Specify species and number: ___________________________ • Animals raised for food production: ☐ Yes ☐ No • History of Toxoplasmosis in animals: ☐ Yes ☐ No ○ Diagnosis method: ☐ Veterinarian ☐ Laboratory ☐ Both Date: /______ Doctor’s Signature: __________________________

PART 2: MEDICAL HISTORY, CLINICAL, AND LABORATORY DIAGNOSIS To be filled out by the attending physician. 1. Physician and Facility Information • Date: /______ • Reporting doctor: ____________________________ • Specialization: ______________________________ • Phone: ___________________ • Email: ___________________ • Region/Province/City: __________________________ • Healthcare facility: ________________________________________________ • Unit/Department: ________________________________________________ 2. Patient Personal Data • Identification code: __________________________ • Surname*: __________________________ • Name*: __________________________ • Date of birth: /______ • Place of birth: __________________ • Gender: ☐ M ☐ F • Nationality: ☐ Italian ☐ Other: ___________ • Arrival in Italy (if foreign): /_____________ • Registered with NHS: ☐ Yes ☐ No • Domicile/Residence: ___________________________________ • Pregnancy: ☐ Yes ☐ No ○ Week of gestation: _______ (***) *An alphanumeric code may be used for anonymity.* 3. Diagnosis of Toxoplasmosis • Confirmed by laboratory: ☐ Yes ☐ No ○ Date: /______ ○ Method: ☐ IFA ☐ EIA ☐ Histology ☐ PCR ☐ MRI ☐ CT • Toxo-Test Result: ☐ Protected ☐ Susceptible ☐ At Risk ☐ Other: _______________ • Attach laboratory report (No.): ________ 4. Clinical and Anamnestic Information • Onset of symptoms: /______ • Place of onset: __________________________ • Clinical features: _____________________________________________ • Previous toxoplasmosis diagnosis: ☐ Yes ☐ No • Co-habitants with Toxoplasmosis: ☐ Yes ☐ No ○ Date of their diagnosis: _____________________ • Concurrent diseases: ☐ Yes ☐ No ○ Specify: _______________________________ • History of blood transfusions or organ transplants: ☐ Yes ☐ No ○ Specify: _______________________________ Clinical diagnosis (check all applicable): • ☐ Acute Toxoplasmosis • ☐ Neurological involvement • ☐ Congenital Toxoplasmosis • ☐ Ocular Toxoplasmosis • ☐ Disseminated infection in immunocompromised subjects Pharmacological treatment: ☐ Yes ☐ No • Specify: _______________________________ 5. Suspected Source of Infection • Food source: _______________________________ • Environmental source: _______________________ • Other: _______________________________ • Presumed period and place of infection: _______________________________ • Date: /________ Doctor’s Signature: __________________________

PART 3: SURVEY ON SOURCES AND CAUSES OF T. GONDII INFECTION Patient Identification Code:_____________________________________________________ To be completed by the official veterinarian. 1. Presumed Foodborne Infection • Presumed contaminated food: ___________________________ • Purchased or consumed at: ___________________________ • Supply chain tracing (include lot number if available): ___________________________ HACCP measures potentially suitable for controlling *T. gondii* contamination (presence of cats, rodents under control, use of potable water, application of adequate hygienic procedures by the staff, environment cleaning and disinfection): (*Check all that apply and provide observations*) 1. ☐ Yes ☐ No–Observations: _______________________________ 2. ☐ Yes ☐ No–Observations: _______________________________ 3. ☐ Yes ☐ No–Observations: _______________________________ Sampling: • Sample 1: ☐ Yes ☐ No ○ Report No.: _______ ○ Date: /______ • Sample 2: ☐ Yes ☐ No ○ Report No.: _______ ○ Date: /______ • Additional samples: ☐ Yes ☐ No ○ Report No.: _______ ○ Date: /______ 2. Presumed Waterborne Infection • Source: ___________________________ • Certification of water potability: ☐ Yes ☐ No • Attach latest analysis certificate: ☐ Yes ☐ No • Water source: ☐ Well ☐ Cistern ☐ Rural irrigation system ☐ Other: ___________________ • Usage: ○ Drinking water as is: ☐ Yes ☐ No ○ Irrigation for vegetables, fruit, hydroponic crops: ☐ Yes ☐ No • Monitoring/measures against pests (insects, rodents, cats near reservoir): ☐ Yes ☐ No • Sampling: ☐ Yes ☐ No ○ Report No.: _______ ○ Date: /______ Notes: ___________________________________________________________ 3. Presumed Environmental Infection • Examination of patient’s animals: ☐ Yes ☐ No ○ Date: /______ ○ Sample type: ___________________ ○ ID code: _______________ ○ Test type: _____________________ ○ Result: ☐ Positive ☐ Negative (*Repeat for additional samples as needed*) • Examination of animal feed: ☐ Yes ☐ No ○ Date: /______ ○ Sample type: ___________________ ○ ID code: _______________ ○ Test type: _____________________ ○ Result: ☐ Positive ☐ Negative • Examination of environmental samples: ☐ Yes ☐ No ○ Date: /______ ○ Sample type: ___________________ ○ ID code: _______________ ○ Test type: _____________________ ○ Result: ☐ Positive ☐ Negative Date: /______ Veterinary Doctor’s Signature: __________________________

PART 4: STRAIN IDENTIFICATION To be completed by Ce.Tox staff 1. Human Biological Samples • Patient ID code: ___________________________ • DNA extraction performed on: ___________________________ • Analytical confirmation and genotyping report No.: ________ (attached) • Reporting doctor: ___________________________ • Ce.Tox Reference: ___________________________ 2. Food Samples • Food sample: ___________________________ • Purpose: Diagnosis of *T. gondii* contamination and strain genotyping • Sampling report No.: _______ • Date of sampling: /______ • Reporting doctor: ___________________________ • Ce.Tox Reference: ___________________________ • Test report (attached) No.: ________ 3. Animal Biological Samples • Sample type: ___________________________ • Animal: ___________________________ • Purpose: Diagnosis of Toxoplasmosis • Sampling report No.: _______ • Date of sampling: /______ • Reporting doctor: ___________________________ • Ce.Tox Reference: ___________________________ • Test report (attached) No.: ________ 4. Conclusions on the Epidemiological Survey Date: /______ Signature of Ce.Tox Head: ___________________________
